# *De novo* RNA-Seq Analysis of the Venus Clam, *Cyclina sinensis*, and the Identification of Immune-Related Genes

**DOI:** 10.1371/journal.pone.0123296

**Published:** 2015-04-08

**Authors:** Baoping Pan, Yipeng Ren, Jing Gao, Hong Gao

**Affiliations:** College of Life Sciences, Tianjin Key Laboratory of Animal and Plant Resistance, Tianjin Normal University, Tianjin, P. R. China 300387; Institute of Oceanology, Chinese Academy of Sciences, CHINA

## Abstract

The Venus clam, *Cyclina sinensis*, is one of the most important bivalves in China. In recent years, increasing expansive morbidity has occurred in breeding areas, imposing significant losses on the national economy. To understand the molecular mechanisms of immune-related genes, we analyzed and sequenced hemolymph samples that were injected with two pathogenic microorganisms using the Illumina Miseq system. After trimming, more than 12 M PE reads with an average length greater than 410 bp were assembled into 70,079 transcripts with a mean length of 980 bp. Using a homology analysis, 102 (135 transcripts) potentially immune-related genes were identified, and most of them exhibited a similar pattern in both samples. These data indicated that the response of the clam to both types of bacterial infection might follow a similar molecular mechanism. Using the TreeFam method, 9,904 gene families and 1,031 unique families of the clam were preliminarily classified in comparison to five related species. A significant number of SSRs were identified, which could facilitate the identification of polymorphisms in Venus clam populations. These datasets will improve our knowledge of the molecular mechanisms driving the immune response to bacterial infection in clam populations and will provide basic data about clam breeding and disease control.

## Introduction

The Venus clam, *Cyclina sinensis*, is one of the most important bivalves in Chinese marine aquaculture. This clam possesses many advantages as a popular seafood, including rapid growth, salt and temperature resistance, pollution tolerance, and a high survival ratio after prolonged removal from the water[1–2]. This clam species is distributed across most of China’s coastline and has a huge consumer market in China, South Korea and Japan. In recent years, increasing morbidity has occurred in breeding areas, imposing significant losses on the national economy. An important cause of increased morbidity is invasion by pathogens[3–4]. Vibrio disease is the most significant cause of death in many marine organisms; the pathogenic taxa mainly include *Vibrio anguillarum*, *Vibrio vulnificus*, *Vibrio alginolyticus* and *Vibrio harveyi*.

*Vibrio anguillarum*, which belongs to the *Vibrionaceae* family and *Vibrio* class, is a common pathogenic microorganism that secretes toxins leading to morbidity in a variety of marine organisms due to hemorrhagic septicemia accompanied by extensive tissue damage and death [5]. Currently, toxins closely related to *Vibrio anguillarum* pathogenicity are widely recognized[6]. Several studies have shown that after a period of clam exposure to low concentrations of *anguillarum* (OD600 = 0.4), a series of active immunological substances, such as acid phosphatase, alkaline phosphatase, superoxide dismutase and lysozyme genes, are significantly upregulated[7–8]. To date, only a few studies have been performed on mussels and oysters.

Minimal studies of bivalve immune factor-related gene expression and regulation (in mussels, oysters and scallops) have been reported. Mytilin B, mytilin C, mytilin D and mytilin G1[9], which are cysteine-rich cationic antimicrobial peptides involved in innate mussel (*Mytilus galloprovincialis*) immunity, have been identified. Using expressed sequence tag (EST) analysis, Gueguen *et*, *al*. screened 20 genes related to immune sequences from a Pacific oyster hemolymph cDNA library and performed an expression experiment on genes induced in response to bacterial infections in oysters[10]. In addition, the scallop heat shock protein 70 and C-type lectin gene have been cloned, and their expression levels have been examined[11–12]. Also in scallop, a selenium-binding cDNA protein was cloned with EST and RACE, and its significant upregulation in the blood cells of scallops after microbial infection was confirmed[13]. However, the immune factor genes have not been identified nor have the system been analyzed in the Venus clam and other bivalves. In this study, we identified the sequences and expression levels of immune-related genes in the Venus clam after bacterial injection. These data may provide a new approach for preventing and treating diseases in bivalves. Transcriptomes are an important resource for the rapid and cost-effective development of genetic markers[14]. Molecular markers derived from the transcribed regions are more conservative, providing greater potential for identifying functional genes. Among the various molecular markers, simple sequence repeats (SSRs) are highly polymorphic, easier to develop and richly diverse[15].

In our study, we used related methods (Data filtering and *de novo* assembly, Gene family comparative and evolutionary analysis and cSSR discovery) from Zhang et al "Transcriptomics and Identification of the Chemoreceptor Superfamily of the Pupal Parasitoid of the Oriental Fruit Fly, *Spalangia endius* Walker (Hymenoptera: Pteromalidae)", PLoS ONE, 2014.

## Materials and Methods

### Ethics statement

No specific permits were required for the sample collection for this study in Tianjin Dagang shoals. The field studies did not involve endangered or protected species. The species in the genus of *Cyclina* are common shellfish and are not included in the “List of Protected Animals in China”.

### Sample collection

*Cyclina sinensis* individuals were collected from the coastal region of Dagang district which location in 117.45°E 38.83°N.", Tianjin, China. and maintained in tanks containing aerated fresh seawater and 5‰ Chlorella sp. for 7 days. The average features were as follows: shell lengths of 28.12 ± 1.49 mm; shell heights of 28.86 ± 1.57 mm; and shell widths of 18.56 ± 0.47 mm. The clams which we used in the experiment was two years old, no damage, no significant difference in morphological index and male and female mixed. The seawater was completely replaced when the density reached 1.020–1.040 g/cm^3^ with a pH of 7.0. The clams were fed 5% chlorella twice per day.

*Vibrio anguillarum* and *Micrococcus luteus* were cultured in a 2216E medium at 28°C for 24 h. The bacterial culture was washed three times with sterile saline. Between each wash, the bacteria were centrifuged at 4,000 ×*g* for 10 min, were resuspended at 1×10^8^ cells/ml in each tube and injected 50ul to each clam about 5×10^6^ cell.

### RNA extraction and library construction for Illumina sequencing

Seventy-two *Cyclina sinensis* were selected at random for the experiment and thirty-six were injected (into the adductor muscles) with 50 μl live *Vibrio anguillarum* resuspended in PBS (OD = 0.4, 1×10^8^ cell/ml), while 36 were injected (into the adductor muscles) with 50 μl live *Micrococcus luteus* (OD = 0.4, 1×10^8^ cell/ml). Total RNA from the clam hemolymph was extracted using TRIZOL (Life Technologies). The quality and quantity of the RNA sample was analyzed and verified using an Agilent 2100 Bioanalyzer (AmershamBiosciences, Uppsala, Sweden) and gel electrophoresis.

Messenger RNAs from the extracted total RNA were isolated using oligo (dT) magnetic beads and were randomly cleaved into short fragments. Next, the first-strand cDNAs were synthesized with random hexamer primers and followed by second-strand cDNA synthesis using DNA polymerase I (New England BioLabs, Ipswich, MA) (NEB) and RNaseH (Invitrogen). The completion of repair, adapter connection and PCR amplification were preformed following a standard protocol (Illumina). Finally, the quality and quantity of the library were verified using an Agilent 2100 Bioanalyzer and an ABI StepOnePlus Real-Time PCR System. The qualified library was sequenced using the Illumina MiSeq platform.

### Data filtering and *de novo* assembly

To ensure the accuracy of the subsequent analysis, a stricter filtering criterion was used to minimize the effects of sequencing errors during gene assembly because sequencing errors will result in additional errors in a short-read assembly algorithm. First, raw reads that had adapter sequences were removed. Then, the low-quality (Q<5) nucleotides at both ends of the reads were trimmed. Finally, the low-quality reads were removed when the average quality score was less than 15 (the Phrap score, which were originally developed by the program Phrap to help in the automation of DNA sequencing in the Human Genome Project and assigned to each nucleotide base call in automated sequencer traces)[16]; single reads less than 36 bp were also removed.

The clean reads were analyzed by Trinity (Release 2013-06-08) for *de novo* assembly using a paired-end model[17]. The following parameters were used in Trinity: min_glue = 1, V = 10, edge-thr = 0.05, min_kmer_cov = 2, path_reinforcement_distance = 150, and group_pairs_distance = 500. Next, any redundant fragments were removed by TGICL (**TGI Clustering tools**) and a Phrap assembler[18]. The parameters were set as follows: a minimum of 95% identity, a minimum of 35 overlapping bases, a minimum of 35 scores and a maximum of 20 unmatched overhanging bases at the sequence ends. Finally, based on sequence similarity, the transcripts were divided into two classes: a cluster (prefixed with 'CL') and a singleton (prefixed with 'unigene'). In a cluster, the similarity between the transcripts was more than 70%[19].

### Functional and expression annotation

BLASTx alignment (E-value <1e^-5^) was performed between the transcripts and the protein databases, which included the NCBI non-redundant protein (Nr) database (last updated in June of 2013), the Swiss-Prot protein database (Release 2013_05), and the Kyoto Encyclopedia of Genes and Genomes (KEGG) pathway database (Release 63.0). The best hits were used to select the transcription direction and CDS (coding region) of one transcript. If the results of different databases conflicted with one another, a prioritization order of Nr, Swiss-Prot, and KEGG was followed when choosing the sequence direction and CDS of a transcript. When a transcript could not predict a CDS using a homology method, the software ESTScan was used for prediction [20]. The peptide sequences were translated using standard codons for CDS with lengths no less than 100 bp. In addition, the transcripts were annotated with an NCBI non-redundant nucleotide (Nt) database (June, 2013) using BLASTn. To annotate the transcripts with GO terms, Blast2GO was used to obtain GO annotations according to the molecular function, biological processes and cellular component ontologies following the Nr BLAST results [21].

BWAaligner (Version 0.7.1) was used to map the reads back to the transcripts using the MEM model with default parameters. Each transcript was normalized into FPKM values (Reads Per kb per Million Fragments)using the best mapped reads, which were determined using the exact-match length, and the read was discarded if it yielded the same results from more than two transcripts[22].

A pathway enrichment analysis for a hawthorn-specific gene was conducted based on an algorithm presented by KOBAS [23], with the entire hawthorn transcriptome set as the background. The P-value was approximated by a hypergeometric distribution test and multiple testing correction using FDR (False Discovery Rate, a set of predictions is the expected percent of false predictions in the set of predictions)[24]. The enriched cutoff was a Q-value less than 0.001.

### Gene family comparative and evolutionary analysis

To identify the gene families and clam-specific genes, we selected the following reference species: *Crassostrea gigas*, *Capitella teleta*, *Lottia gigantea*, *Helobdella robusta* and *Strongylocentrotus purpuratus* [25]. For comparative analysis, we used the fellow pipeline to cluster individual genes into gene families using TreeFam [26]. First, we collected protein sequences longer than 33 amino acids from these six species. The longest protein isoform was retained from each gene. Based on the clam genes derived from the mRNA-seq, putative alternative splice variants were filtered based on sequence similarity using an overlap ratio criteria of no less than 70% for any of the sequences. Furthermore, the longest protein isoform was retained for each cluster. Second, Blastp was used for all of the protein sequence alignments against a database containing a protein dataset of all of the species with an e-value of 1e-7. Next, fragmental alignments for each gene pair were conjoined using Solar [27]. Third, the gene families were extracted with Hcluster using the default parameters. Single-copy ortholog genes were used to construct a phylogenetic tree using PhyML with default parameters [19,28–29].

### cSSR discovery

The cSSRs (*Cyclina sinensis* Simple Sequence Repeats) were identified using a Perl script from MIcroSAtellite (MISA) with transcripts for reference. Mono-, di-, tri-, tetra-, penta- and hexa-nucleotide sequences with a minimum number of 12, 6, 5, 5, 4 and 4 repeats, respectively, were applied as the search criteria (http://pgrc.ipk-gatersleben.de/misa/). Primer 3–2.3.4 was used to design the PCR primers using the default settings. Primers were filtered based on the following criteria: (1) no SSRs existed in the primer; (2) three mismatches for the 5’-site and one mismatch for the 3’-site were allowed to align the primers to the transcripts; (3) each primer could only be mapped to one transcript [19,30].

## Results and Discussion

### Sequencing and *de novo* assembly

A cDNA library for the *C*. *sinensis* clam was generated using the mRNA-Seq on an Illumina Miseq platform. Using the Illumina paired-end sequencing model, each sequencing feature could yield 2 × 250 bp independent reads from both ends of each fragment. After the data were trimmed, a total of 6,694,694 high-quality reads (2,808,369,022 base pairs) from sample M stimulated by *Vibrio anguillarum* and 5,483,161 high-quality reads (2,314,488,797 base pairs) from sample T stimulated by *Micrococcus luteus* were obtained. The average length of the reads was more than 200 Nt (Table 1).

**Table 1:** Summary of sequencing and assembly for the Venus clam, *Cyclina sinensis*.

Reads	Sample T	Sample M
Mean length (bp)	217+205	216+203
Total number*	5,483,161(87.49%)	6,694,694(86.79%)
Total bases (bp)	2,314,488,797	2,808,369,022
Q20 percentage	97.38%	97.96%
**All Transcripts** (≥250 bp)		
Total number	70,079	
Total length (bp)	68,712,488	
Unigene number	59,019	
N50 length (bp)	1,670	
Mean length (bp)	980	
ORFs/CDS	35,103	

* indicates the proportion of reads that could be mapped to transcripts.

**Fig 1:**
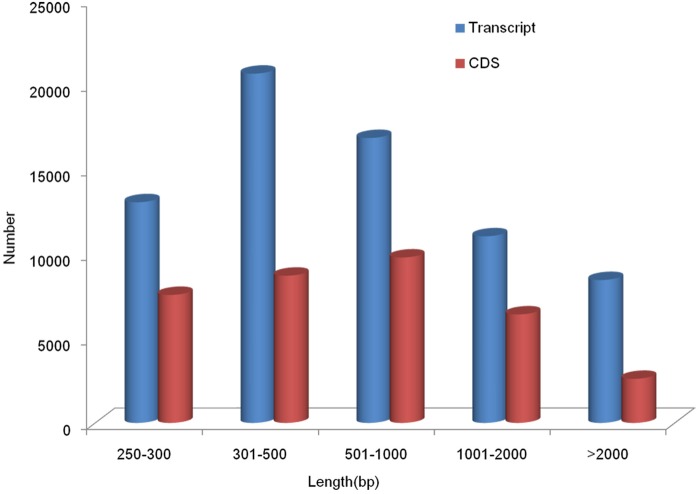
Size distribution of the transcripts and CDSs of the Venus clam, *Cyclina sinensis*. The blue and red bars indicate transcripts and CDS, respectively.

The clean reads from samples T and M were pooled for *de novo* assembly using Trinity [17]. Finally, a total of 70,079 transcripts longer than 250 bp were generated with a mean length of 980 bp and an N50 of 1,670 bp (Table 1). This assembly produced a substantial number of large transcripts: 36,355 transcripts (51.88%) longer than 500 bp, 19,496 transcripts (27.82%) longer than 1,000 bp and 8,455 transcripts (12.65%) longer than 2,000 bp (Fig 1). Using the BWA aligner, 87.49% (in M) and 86.79% (in T) of the reads were realigned to the transcripts, and the transcript coverage was positively related to the length of the given transcripts. The average coverage of all of the transcripts was 75-fold.

### Annotation and CDS prediction

For annotation, the homologous sequences of the transcripts were searched from several databases, including the Nr, Nt, UniProt/Swiss-Prot and KEGG, using the Basic Local Alignment Search Tool (BLAST) with an E-value threshold of 1e-5. A total of **30,930** (44.14%) transcripts were annotated to at least one of the four databases (S1 Table). Among them, 29,219 transcripts were annotated to the Nr database, and 49.52% (14,468) of the annotated transcripts were homologous with *Crassostrea gigas*, followed by *Capitella sp*. I (2315/7.92%), *Saccoglossus kowalesvskii* (1219/4.17%), *Amphioxus floridae* (1191/4.08%) and *Strongylocentrotus purpuratus* (890/3.05%). Additionally, 23,344 transcripts had significant matches in the Swiss-Prot database, 20,455 had homologous sequences in the KEGG database, and only 7,739 had a BLASTn match in the Nt database (Table 2). Based on the Nr annotation, 12,225 sequences were classified in GO.

**Table 2:** Summary of annotations of the transcripts from the Venus clam, *Cyclina sinensis*.

Annotated databases	NO. of hits
NT	7,739
NR	29,219
UniProt/Swiss-Prot	23,344
KEGG	20,455
GO	12,225
**Total**	**30,930**

In total, 20,455 transcripts were assigned to 259 pathways in the KEGG database (S2 Table). Metabolic pathways (2889 transcripts), regulation of the actin cytoskeleton (988 transcripts) and focal adhesion (941 transcripts) were the most represented pathways. Furthermore, 15 immune system pathways were identified, including the T-cell receptor signaling pathway, the B-cell receptor signaling pathway, the Toll-like receptor signaling pathway and the NOD-like receptor-signaling pathway. In total, 2660 transcripts were annotated to 506 enzymes, which catalyzed the most enzymes in the immune system pathways. These enzymes expressed an average of 5 transcripts, suggesting that these genes were more actively transcribed in the experimental sample.

The CDS (coding sequences) were predicted using BLASTx and ESTscan. Using homologous matches, 29,877 transcripts were identified as CDS, with mapping lengths no less than 100 bp (Fig 1). Other transcripts were processed with ESTScan, and 5,226 transcripts were detected with coding regions no less than 100 bp. The transcripts without identified coding regions were likely to be too short to meet the criterion for CDS prediction. Another possibility is that some of these transcripts might have been non-coding RNAs. A similar result has been reported in other studies [31–32]. These putative non-coding RNAs must be validated in a future study.

### Immune gene identification and expression

**Fig 2:**
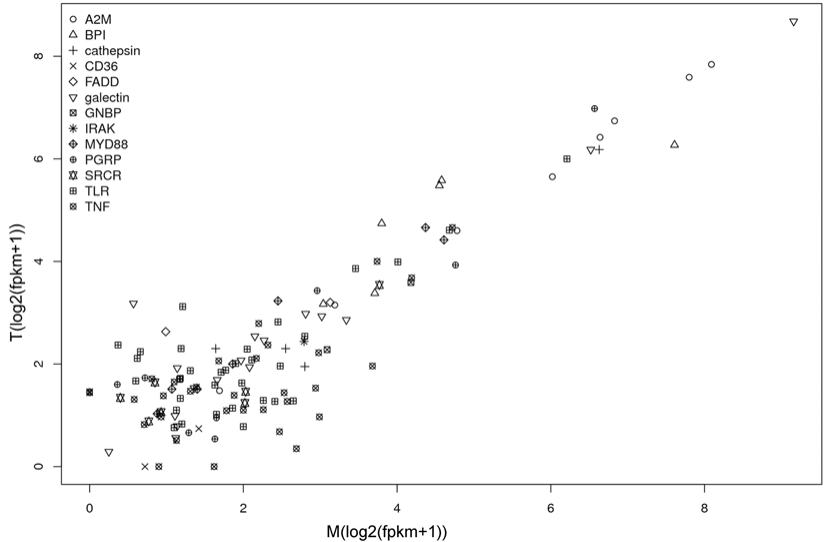
The expression patterns of immune-related genes in sample M and sample T of the Venus clam, *Cyclina sinensis*. The selected immune-related genes include alpha-2-macroglobulin(A2M), bactericidal permeability-increasing protein (BPI), cathepsin, leukocyte differentiation antigen (CD36), FAS-associated death domain protein (FADD), galectin, GNBP, interleukin-1 receptor-associated kinase (IRAK), Myd88, peptidoglycan recognition protein (PGRP), scavenger receptor cysteine-rich protein (SRCR), perforin-related protein, toll-like receptor (TLR) and tumor necrosis factor (TNF).

To understand the immune system, 102 (135 transcripts) immune-related genes were identified, including 5 (8) alpha-2-macroglobulin (A2M), 5 (6) bactericidal/permeability-increasing protein (BPI), 4 cathepsin, 2 CD36 antigens, 2 FAS-associated death domain proteins (FADD), 6 (17) galectin, 2 GNBPs, 1 interleukin-1 receptor-associated kinase (IRAK), 4 (7) Myd88, 7 (8) peptidoglycan recognition proteins (PGRP), 6 (12) scavenger receptor cysteine-rich proteins (SRCR), 26 (32) tumor necrosis factors (TNF), and 32 (34) toll-like receptors (TLR) (Fig 2 and S2 Table). In these genes, the average expression levels in M and T were 17.2 and 14.2, respectively, or approximately 1.7 and 1.4 times the average expression of the whole transcripts (the average expression of all of the transcripts in samples M and T: 9.9 and 10.0 FPKM). Most of these genes have a high expression level, with at least one gene or transcript in the gene family highly expressed (over the average expression), such as A2M, BPI, caprin, galectin, GNBP, Myd88, PGRP, SRCR, TNF, and TLR. The highest expression level (569FPKM) belonged to galectin (Unigene9462_sinensis). This finding suggests that this gene may be actively expressed in vivo, which is consistent with previous studies[8,17]. Most of the sequences have a considerable expression level in both M and T, except for 11 transcripts that are differentially expressed between M and T (Fig 2). This result suggests that the clam response to both gram-negative bacteria and gram-positive bacteria follows a similar molecular mechanism.

### Gene expression and differential expression of genes

The 1,000 most abundant transcripts in each sample were analyzed. After being merged, a total of 1,289 transcripts were obtained and the expression value of the least abundant transcript was 67.6 FPKM. These data suggest that most transcripts were highly expressed in both M and T. A pathway enrichment analysis indicated that most of these transcripts were enriched in 28 pathways and that they could be divided into four classes. The first class included ribosome, antigen processing and presentation and oxidative phosphorylation. After injection, the clam response to stress produced by injection requires a large amount of active protein biosynthesis and modification, and these three pathways provided the basic raw materials and modified the antibody process. The second class included pathways that respond to pathogenic *Escherichia coli* infection, *Salmonella* infection, shigellosis, pertussis, *Staphylococcus aureus* infection, legionellosis and bacterial invasion of epithelial cells. These are human diseases, which are caused by bacterial infection. This finding suggests that the clam possesses an immune system homologous to a human for defense against bacterial attacks. The third class contained immune system pathways, such as those related to complementary and coagulation cascades, leukocyte transendothelial migration, and phagosomes; these processes are part of the animal defense system. The last class included other human diseases and other pathways. Above all, nearly half (12/28) of the enriched pathways were related to bacterial infection and immune response, suggesting that the immune response to pathogenic microorganisms is very active in-vivo, which is consistent with previous research (Table 3).

**Table 3:** Summary of the immune-related pathways in *Cyclina sinensis*.

Immune-related pathways	Unigene number	P-value	Q-value	Contain differential expression
Hematopoietic cell lineage	37	0.03405	0.06154	YES
Complement and coagulation cascades	48	0.002213	0.007191	YES
Toll-like receptor signaling pathway	115	0.000525	0.002387	YES
NOD-like receptor signaling pathway	100	0.000535812	0.002387217	NO
RIG-I-like receptor signaling pathway	79	0.015546	0.032031	YES
Cytosolic DNA-sensing pathway	79	0.004385461	0.012355324	NO
Natural killer cell mediated cytotoxicity	97	0.021219	0.041481	YES
Antigen processing and presentation	76	2.07E-08	9.14E-07	YES
Fc epsilon RI signaling pathway	83	0.00756	0.018103	YES
Fc gamma R-mediated phagocytosis	129	5.00E-06	5.32E-05	YES
Leukocyte transendothelial migration	143	7.78E-05	0.000523	YES
Chemokine signaling pathway	171	0.000791	0.003225	YES

In total, 3,665 genes representing 5.23% (3,665/70,079) of the total putative transcripts were differentially expressed between the M and T (twofold or more change and an FDR<0.001). Among them, 2,321 genes were upregulated, and 1,343 genes were downregulated in T (S3 Table). Only a few immune-related genes were differentially expressed between these two cases. This finding indicates that clam response to both gram-negative bacteria and gram-positive bacteria may involve a similar molecular mechanism.

### Gene family comparative and evolutionary analysis

**Fig 3:**
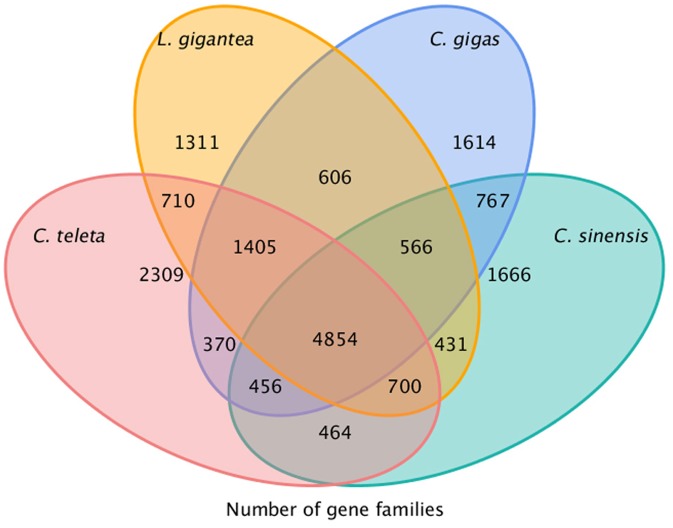
Summary of the gene family classification of four related species, *Cyclina sinensis*, *Crassostrea gigas*, *Lottia gigantea* and *Capitella teleta*. Only putative peptides were used in *Cyclina sinensis*, while whole peptides were used in the other species.

Using TreeFam and the pipeline described in the methods, we obtained 9,904 gene families and 1,031 unique families from *C*. *sinensis* and the five reference genomes. The common and unique gene families among *C*.*sinensis*, *Crassostrea gigas*, *Lottia gigantea* and *Capitella teleta* are summarized in Fig 3. The results revealed that, compared with other species, *C*. *gigas* shared the most gene families (6,643/67.07%) with *C*. *sinensis*. Based on the gene family analysis, a significant percentage of transcripts (56.21% / 14,173) in the clam were found to not be from a conserved lineage, which might be due to the presence of novel families. Alternatively, the assembled transcripts may be from non-conserved protein areas where homology or assembly errors are not detected, which is in agreement with several other papers [31,33–34]. The result of the KEGG enrichment is summarized in S4 Table. Interestingly, two signaling molecules and interactions (ECM-receptor interaction, neuroactive ligand-receptor interaction) and three translation pathways (RNA transport, mRNA surveillance pathway, and ribosome biogenesis in eukaryotes) were enriched, which suggests that a large amount of antibody proteins were biosynthesized after macro-organism infection. Additionally, two carbohydrate metabolism pathways (starch and sucrose metabolism, galactose metabolism) may provide a large amount of energy at this stage, which is supported by previous research.

**Fig 4:**
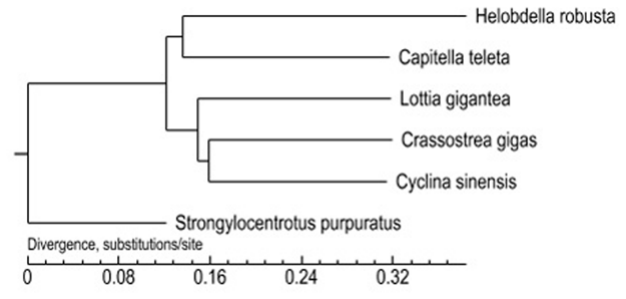
Phylogenetic tree showing the evolutionary relationship of the Venus clam, *Cyclina sinensis*, to four closely related species (*C*. *gigas*, *C*. *teleta*, *L*. *gigantea*, *H*. *robusta*), based on RNA-seq data. The out group was *S*. *purpuratus*. Single copy gene families (1,343 genes comprising 873,976 peptide sites) were concatenated and used in the program PhyML with default parameters.

To understand the evolutionary relationship between *C*. *sinensis* and other marine animals, a set of 1,343 single copy gene families were obtained by the TreeFam method and were concatenated into a super-peptide (873,976 peptide sites) for constructing a phylogenetic tree using PhyML; *S*. *purpuratus* was used as the out group (Fig 4). *C*. *sinensis* and *C*. *gigas* belong to Bivalvia, and *L*. *gigantea* are from Mollusca. *C*. *teleta* and *H*. *robusta* are *Annelida*, and all five belong to *Lophotrochozoa*. Our tree is consistent with current classification. Molecular evidence is considered more effective for taxonomic classification in comparison with other existing methods. The transcriptome data and mitochondrial genome for phylogeny and orthology analysis have become increasingly popular[35–37]. In particular, certain taxons, such as arthropods, echinoderms, tetrapods and snakes, possess radical transformations, both morphologically and physiologically [38].

### cSSR discovery

To detect new molecular markers, all of the transcripts were used to mine potential cSSR (cDNA Simple Sequence Repeat) motifs using the MISA Perl script (http://pgrc.ipk-gatersleben.de/misa/). In total, 3,222 (4.6%) transcripts contained 3,594 cSSRs, from which **1,409** tri-nucleotide (39.20%) and **1,233** mono-nucleotide (34.31%) repeat motifs had the highest frequencies (Table 4). After designing and filtering the primers, 612 cSSR markers were found to have at less one primer (S5 Table). These data may better elucidate clam polymorphism.

**Table 4:** The lengths of identified cSSRs based on the number of repeat units.

Number of repeats	Mono-nucleotide	Di-nucleotide	Tri-nucleotide	Quad-nucleotide	Penta-nucleotide	Hexa-nucleotide
4	-	-	-	-	42	4
5	-	-	935	118	0	0
6	-	396	333	21	0	0
7	-	155	122	0	0	0
8	-	71	19	0	0	0
9	-	42	0	0	0	0
10	-	37	0	0	0	0
11	-	60	0	0	0	0
12	410	6	0	0	0	0
13	229	0	0	0	0	0
14	230	0	0	0	0	0
15	123	0	0	0	0	0
>15	241	0	0	0	0	0
SubTotal	1,233	767	1,409	139	42	4

## Conclusions

This study aimed at advancing basic molecular knowledge of immune-related genes in clam. Several interesting results were obtained: 1) a large number of candidate genes potentially involved in immune-related genes include its pathways were identified, and the expression data suggested that clam response to several types of bacteria may function in a similar molecular mechanism; 2) orthologous and unique sequences were preliminary classified by comparisons to relative species; and 3) a significant number of SSRs were identified, which may facilitate the identification of polymorphisms in Venus clam populations. These datasets will improve our understanding of the molecular mechanisms of immune responses to bacterial infection in clams.

## Supporting Information

S1 Table Summary of the transcript annotations from the public databases. (XLS)

S2 Table Key immune-related genes identified in the Venus clam. (XLS)

S3 Table List of differentially expressed genes. (XLS)

S4 Table KEGG pathway enrichment of clam-specific genes. (XLS)

S5 Table Primer information for cSSR. (XLS)

## References

[1] Wang HZ. Zhejiang fauna. Molluscs articles.1991.

[2] Wang RC, Wang ZP, Zhang JZ. Shellfish Aquaculture Science Qing Dao, Qingdao Ocean University Press 1993: 208–209.

[3] Cao H. Coastal beach clam Preliminary cause of death and Countermeasures. Scientific Fish Farming. 2004;6(6):403–412.

[4] Liang YB, Yang B, Wang LJ, Zhang XC, Wu ZQ, Jiang YW, et al Disease triggering mechanism and protecting policy of the mariculture shellfish in coast of Yellow Sea. Marine Environmental Science.2000;19: 5–10.

[5] Ge L, Huang J, Li Q. Advance in Studies on Virulence Genes of *Vibrio anguillarum* . Microbiology.2007;34(3):584–586.

[6] Wang HB, Cheng M, Wu Q, Li SH, Yan BL. Changes of Some Immunoactive Enzymes in *Charybdis japonica* Infected with *Vibrio parahemolyticus* . Journal of Hydroecology.2012;3:330–331.

[7] Pan BP, Song X, Luo KY, Ge DY, Gao WW. Expression of lysozyme gene in *Vibrio anguillarum* challenged *Cyclina sinensis* . Oceanologia ET Limnologia Sinica.2010;41(6): 901–906.

[8] Liu KJ, Liu Y, Pan BP. Effect of Vibrio anguillarum on Activity of Lysozgme and Superoxide Dismutase in *Cyclina sinensis* . Sichuan Journal of Zoology. 2011;30(5): 802–804.

[9] Mittapalli O, Bai X, Mamidala P, Rajarapu SP, Bonello P. Herms DATissue-specific transcriptomics of the exotic invasive insect pest emerald ash borer (*Agrilus planipennis*). PLoS one.2010;5(10): 13708. 10.1371/journal.pone.0013708 PMC2965670 21060843

[10] Gueguen Y, Cadoret JP, Flament D, Barreau-Roumiguiere C, Girardot AL, Garnier J, et al Immune gene discovery by expressed sequence tags generated from hemocytes of the bacteria-challenged oyster, *Crassostrea gigas* . Gene.2003;303: 139–145. 12559575 10.1016/s0378-1119(02)01149-6

[11] Song LS, Wu LT, Ni DJ, Chang YQ, Xu W, Xing KZ.The cDNA cloning and mRNA expression of heat shock protein 70 gene in the haemocytes of bay scallop (*Argopecten irradians*, Lamarck 1819) responding to bacteria challenge and naphthalin stress. Fish & Shellfish Immunol.2006;21:335–345. 16530426 10.1016/j.fsi.2005.12.011

[12] Zhu L, Song Linsheng, Xu W, Qian PY. Molecular cloning and immune responsive expression of a novel C-type lectin gene from bay scallop *Argopecten irradians* . Fish & Shellfish Immunol.2008;25:231–238. 18640058 10.1016/j.fsi.2008.05.004

[13] Song LS, Zou HB, Yaqing Chang, Xu W, Wu LT. The cDNA cloning and mRNA expression of a potential selenium-binding protein gene in the scallop *Chlamys farreri* . Dev.Comp. Immunol.2006;30:265–273. 15975653 10.1016/j.dci.2005.04.001

[14] Du HX, Bao ZM, Hou R, Wang S, Su HL, Yan JJ, et al Transcriptome sequencing and characterization for the sea cucumber *Apostichopus japonicus* (Selenka, 1867). PLoS One.2012;7: 33311. 10.1371/journal.pone.0033311 PMC3299772 22428017

[15] Garg R, Patel RK., Tyagi AK, Jain M. De novo assembly of chickpea transcriptome using short reads for gene discovery and marker identification. DNA Res.2011;18: 53–63. 10.1093/dnares/dsq028 21217129 PMC3041503

[16] Ewing B, Hillier L, Wendl MC, Green P. Base-calling of automated sequencer traces using phred. I. Accuracy assessment. Genome Res. 1998;8 (3): 175–185. 9521921 10.1101/gr.8.3.175

[17] Grabherr MG, Haas BG, Yassour M, Levin JZ, Thompson DA, Amit I, et al Full-length transcriptome assembly from RNA-Seq data without a reference genome. Nat Biotechnol.2011;29: 644–652. 10.1038/nbt.1883 21572440 PMC3571712

[18] Pertea G, Huang X, Liang F, Antonescu V, Sultana R, Karamycheva S, et al TIGR Gene Indices clustering tools (TGICL): a software system for fast clustering of large EST datasets. Bioinformatics.2003;19: 651–652. 12651724 10.1093/bioinformatics/btg034

[19] Zhang YP, Zheng Y, Li DS, Fan YL. Transcriptomics and Identification of the Chemoreceptor Superfamily of the Pupal Parasitoid of the Oriental Fruit Fly, Spalangia endius Walker (Hymenoptera: Pteromalidae). PLoS one.2014;9(2). 10.5339/gcsp.2014.19 24505315 PMC3914838

[20] Iseli C, Jongeneel CV, Bucher P. ESTS can: a program for detecting, evaluating, and reconstructing potential coding regions in EST sequences. Proc Int Conf Intell Syst Mol Biol.1999:138–148. 10786296

[21] Conesa A, Götz S, García-Gómez JM, Terol J, Talón M, Robles M. Blast2GO: a universal tool for annotation, visualization and analysis in functional genomics research. Bioinformatics.2005;21: 3674–3676. 16081474 10.1093/bioinformatics/bti610

[22] Mortazavi A, Williams BA, McCue K, Schaeffer L, Wold B. Mapping and quantifying mammalian transcriptomes by RNA-Seq. Nat Methods.2008;5: 621–628. 10.1038/nmeth.1226 18516045 PMC13303166

[23] Xie C, Mao X, Huang J, Ding Y, Wu J, Dong S, et al KOBAS 2.0: a web server for annotation and identification of enriched pathways and diseases. Nucleic Acids Res.2011;39: 316–322. 10.1093/nar/gkr483 PMC3125809 21715386

[24] Benjamini Y, Yekutieli D. The control of the false discovery rate in multiple testing under dependency. The Annals of Statistics. 2001;29: 1165–1188.

[25] Simakov O, Marletaz1 F, Cho SJ, Eric EG, Havlak P, et al Insights into bilaterian evolution from three spiralian genomes. Nature.2013;493 (24): 526–531. 23254933 10.1038/nature11696 PMC4085046

[26] Li H, Coghlan A, Ruan J, Coin LJ, Hériché JK, Osmotherly L. TreeFam: a curated database of phylogenetic trees of animal gene families. Nucleic Acids Res.2006;34: 572–580. 10.1093/nar/gkj118 PMC1347480 16381935

[27] Yu XJ, Zheng HK, Wang J, Wang W, Su B. Detecting lineagespecific adaptive evolution of brain-expressed genes in human using rhesus macaque as outgroup. Genomics.2006;88: 745–751. 16857340 10.1016/j.ygeno.2006.05.008

[28] Guindon S, Gascuel O. A simple, fast and accurate algorithm to estimate large phylogenies by maximum likelihood. Syst. Biol.2003;52: 696–704. 14530136 10.1080/10635150390235520

[29] Guindon S, Dufayard J-F, Lefort V, Anisimova M, Hordijk W, Gascuel O. New Algorithms and Methods to Estimate Maximum-Likelihood Phylogenies: Assessing the Performance of PhyML 3.0. Syst Biol.2010;59(3): 307–321. 10.1093/sysbio/syq010 20525638

[30] Untergasser A, Cutcutache I, Koressaar T, Ye J, Faircloth BC, Remm M, et al Primer3—new capabilities and interfaces. Nucleic Acids Res.2012;40:115. 10.1093/nar/gks596 PMC3424584 22730293

[31] Bai X, Mamidala P, Rajarapu SP, Jones SC. Mittapalli O Transcriptomics of the Bed Bug (*Cimex lectularius*). PLoS ONE. 2011;6 (1): 16336. 10.1371/journal.pone.0016336 PMC3023805 21283830

[32] Liu S, Li W, Wu Y, Chen C, Lei J. De Novo Transcriptome Assembly in Chili Pepper (Capsicum frutescens) to Identify Genes Involved in the Biosynthesis of Capsaicinoids. PLoS one.2013;8(1): 48156 10.1371/journal.pone.0048156 23349661 PMC3551913

[33] Wang JP, Lindsay BG, Leebens-Mack J, Cui L, Wall K, Miller WC. Pamphilis CW EST clustering error evaluation and correction.Bioinformatics.2004;20: 2973–2984. 15189818 10.1093/bioinformatics/bth342

[34] Liang H, Carlson JE, Leebens-Mack JH, Wall PK, Mueller LA, Buzgo M, et al An EST database for Liriodendron tulipifera L. floral buds: the first EST resource for functional and comparative genomics in Liriodendron. Tree Genet Genomes.2008;4: 419–433.

[35] Castoe TA, Gu W, de Koning APJ, Daza JM, Jiang CL, Parkinson DD. Dynamic nucleotide mutation gradients and control region usage in squamate reptile mitochondrial genomes. Cytogenet Genome Res. 2009;127(2–4):112–127. 10.1159/000304046 20215734 PMC2872679

[36] Meusemann K, Reumont BM, Simon S, Roeding F, Strauss S, Kück P, et al A Phylogenomic Approach to Resolve the Arthropod Tree of Life. Mol Biol Evol.2010;27(11): 2451–2464. 10.1093/molbev/msq130 20534705

[37] Kocot KM, Cannon JT, Todt C, Citarella MR, Kohn AB, Meyer A, et al Phylogenomics reveals deep molluscan relationships. Nature.2011;477:452–456. 10.1038/nature10382 21892190 PMC4024475

[38] Carroll RL. Patterns and processes of vertebrate evolution. Cambridge Univ. Press, New York 1997.

